# Supplementary material for: Barriers and Facilitators of Online Grocery Services: Perceptions from Rural and Urban Grocery Store Managers
Source: Nutrients. 2022 Sep 14;14(18):3794. doi: 10.3390/nu14183794 (PMC9502010; doi:10.3390/nu14183794)
Supplement: Supplementary file 1 [file nutrients-14-03794-s001.zip › nutrients-1905115-supplementary.pdf]

nutrients

Supplementary Materials

Barriers and Facilitators of Online Grocery Services: Perceptions from Rural and Urban Grocery Store Managers

Online Grocery Store Manager Interview Guide

General Questions about Benefits and Challenges to Online Shopping:

1. First, can you tell me how long you've been offering online ordering for your customers? How did your store first become interested in offering online ordering services?
2. *Probe:* Was this a corporate or community driven initiative? What was it like to get that process started at your store?
3. (*SNAP Stores follow-up question*): How long have you accepted SNAP EBT benefits online? Can you tell me about that process?
4. Can you talk about operationally from your end, what it looks like when the store receives an online order from a customer until groceries are delivered or picked up by the customer?
5. Do you use a third-party platform for your store's online ordering? Can you talk about your experience using this third-party system for online shopping?
6. From your perspective what have the benefits of online ordering been for your store in general? What about you as the manager of the store? What about for your employees?
7. Have you heard directly from any of your customers about what they think are the benefits of grocery shopping online?
8. Has your store experienced challenges fulfilling online orders between when they are placed and when they are picked up or delivered?
9. What challenges or frustrations have you heard from your store staff related to fulfilling online orders?
10. Have you heard directly from customers about any challenges with online shopping? If so, can you give me some examples?
11. What do you think could help improve online shopping fulfillment/process for store employees fulfilling orders? For customers?

Barriers related to Product Selection:

12. For online shopping, we've heard that customers want more control over the types of produce or meat that are selected or the items that are substituted. Is there a way that customers can have more control with this process at your store?

Online Platform Questions:

13. How much control, if any, do you have over that website?
14. Who in the store oversees the website for online purchases?
15. Do you find it difficult to match up the website features for online with fulfilling orders?
16. What could be improved with the online website from your perspective?

Barriers related to Cost:

17. Can you tell us about how prices are set online? (i.e. Is this process consistent with in-store pricing strategies?)
18. Are there deals for in-store items that aren't found online and vice versa?
19. What kind of fees are associated with online ordering in general? Are there any order minimums, delivery fees, etc.?
20. Are these fees the same for all customers? What would be needed on your end for these fees to be waived for SNAP customers?
21. In regard to curbside pick-up rather than delivery, are there any fees and/or service charges associated with this service? Can you talk a little bit about curbside in general?
22. Probe if not captured previously: Operationally what does it look like from the store side?
23. Probe if not captured previously: Can SNAP customers utilize this service? If not, what would be needed on your end to expand this to SNAP customers?

Rural Grocery Store Questions Only:

24. Grocery delivery services may be more challenging in rural communities due to longer distances between the warehouse and customers. Is providing grocery delivery in rural parts of your community a challenge for you? What would help you expand delivery services from your store?
25. What are the barriers to utilizing more delivery options?
26. If delivery is not offered by store: If home delivery was offered as an option, do you think shoppers would utilize it?
27. In your opinion, what is the best way to expand delivery services to shoppers?

SNAP Questions for Stores Currently Accepting SNAP-EBT Online:

28. In your opinion, what could help make the online grocery shopping ordering platform easier for SNAP shoppers? Either for the store or your employees?
29. When transitioning to accepting online SNAP orders for your store, what was most challenging or difficult?

Questions for Stores Not Currently Accepting SNAP Online:

30. Do you know why your store doesn't accept SNAP online benefits?
31. Would it be valuable for your store to offer online ordering options to SNAP customers specifically, where orders could be placed online and picked up or delivered? Why/why not?
32. What support would your store need to navigate the process of transitioning to accepting online SNAP orders?

Brick and Mortar Grocery Store Managers Interview Guide

General Questions About Brick-and-Mortar Grocery Shopping:

1. Can you talk a little bit about what the last 18-months looked like for your store and what some of your biggest takeaways were?
2. Probe: What are some lessons learned managing a grocery store during a pandemic?
3. Probe: What, if anything, are you changing about your store now?

Perspectives of Online Grocery Shopping:

4. We're seeing more and more stores begin to offer online shopping. What are your thoughts on online grocery services?

5. How do you think your customers would respond if this store started offering online grocery services? Would certain kinds of customers use online services more than others?
6. Compared to online shopping what are some benefits for your customers/store/employees of traditional in-store shopping?
7. What support would your store need to navigate the process of transitioning to offer online ordering? Would this be feasible operationally for your store?

Expansion of SNAP-EBT:

8. What support would you need to offer SNAP/EBT online services?
9. Do you think it would be valuable for your store to offer online ordering options to SNAP customers specifically, where orders could be placed online and picked up or delivered? Why/why not?
10. Probe: What do you anticipate the positives or negatives would be, and if you were to offer this to your customers? To SNAP customers?
11. Probe: Would your store have the capacity and capability to offer this at this time?

Perspectives of Local Community:

12. How long has your store been in business in this community?
13. What changes have you seen since you started in your position as manager? What things have stayed the same?
14. In your experience how have online retailers that offer grocery pick-up and/or delivery changed your business?
15. In your experience how has the introduction of more dollar type stores changed your business? Your community?
16. What do you see as the “future” for in-store or traditional brick and mortar grocery stores? For your store, specifically?
